# Activation of *K-RAS *by co-mutation of codons 19 and 20 is transforming

**DOI:** 10.1186/1750-2187-6-2

**Published:** 2011-03-03

**Authors:** Adam Naguib, Catherine H Wilson, David J Adams, Mark J Arends

**Affiliations:** 1 Medical Research Council Dunn Human Nutrition Unit, Wellcome Trust/MRC Building, Cambridge, CB2 0XY, UK; 2 Wellcome Trust Sanger Institute, Wellcome Trust Genome Campus, Hinxton, Cambridge, CB10 1SA, UK; 3 Department of Pathology, University of Cambridge, Addenbrooke's Hospital, Cambridge, UK, CB2 0QQ, UK

## Abstract

The *K-RAS *oncogene is widely mutated in human cancers. Activating mutations in *K-RAS *give rise to constitutive signalling through the MAPK/ERK and PI3K/AKT pathways promoting increased cell division, reduced apoptosis and transformation. The majority of activating mutations in *K-RAS *are located in codons 12 and 13. In a human colorectal cancer we identified a novel *K-RAS *co-mutation that altered codons 19 and 20 resulting in transitions at both codons (L19F/T20A) in the same allele. Using focus forming transformation assays *in vitro *, we showed that co-mutation of L19F/T20A in *K-RAS *demonstrated intermediate transforming ability that was greater than that of individual L19F and T20A mutants, but less than that of G12D and G12V *K-RAS *mutants. This demonstrated the synergistic effects of co-mutation of codons 19 and 20 and illustrated that co-mutation of these codons is functionally significant.

## Findings

Mutations in *RAS *family genes occur in approximately 20-30% of all human cancers, with mutations in the *K-RAS *gene comprising ~80% of these mutations [1]. *K-RAS *mutations have been documented in the majority of human cancer types with pancreatic (~90% of these cancers) and colorectal (~40%) cancers demonstrating the highest incidence of mutations in this gene [2,3]. *K-RAS *codons 12 and 13 are the most common sites of oncogenic activation with over 90% of documented mutations being found in these codons [4]. Amino acid alterations at these codons, which encode amino acids adjacent to the GDP/GTP binding pocket, reduce or abolish GTPase activity of K-RAS after GAP binding and lock the protein in an active, GTP-bound state [5]. Codons 12 and 13 in wildtype *K-RAS *both encode glycine residues. The incorporation of other amino acids, most commonly aspartate and valine at codon 12 and aspartate at codon 13 [6], brings about projection of larger amino acid side chains into the GDP/GTP binding pocket of the protein, interfering with the geometry of the transition state in which GTP hydrolysis is catalysed [7]. Mutations in codons 61 and 146 have also been described to be oncogenic in *K-RAS*, although mutations at these positions occur at a much lower prevalences (<5% of total *K-RAS *mutations) than codon 12 and 13 mutations [4]. Activation of *RAS *has several effects on rodent cells *in vitro*, including the establishment of a transformed phenotype with anchorage independent growth in soft agar, transformed focus formation, as well as tumour formation following injection into animals [8-10]. Our previous mouse model studies have shown that induced expression of mutant *K-RAS *accelerates intestinal adenoma formation *in vivo *on both a mutant *Apc *background and a *Msh2*-null background, increases proliferation, modulates apoptosis and alters gene expression patterns [11-15]. In this study, we identify a novel double mutation outside of codons 12 and 13, recoding *K-RAS *codon 19 from leucine to phenylalanine (L19F) and codon 20 from threonine to alanine (T20A), in a colorectal cancer and we demonstrate that it causes transformation *in vitro *and is therefore a rare, but functionally significant co-mutation of *K-RAS*.

**Figure 1:**
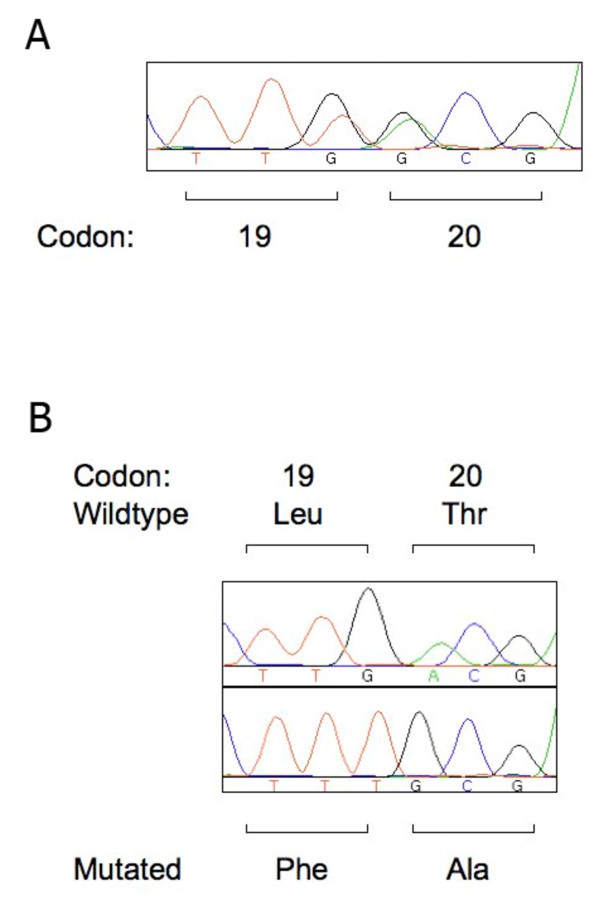
**Dideoxysequence traces of the L19F and T20A double mutation in *K-RAS***. (A) Identification of double peaks at the third base of codon 19 and first base of codon 20 in a colorectal cancer. This sequence trace did not determine whether both base changes were on the same or two independent alleles. Harvesting of tumour genomic DNA and sequencing methodology has been described in detail elsewhere [16]. (B) Sequence trace (upper panel), demonstrating the normal leucine and threonine codons at positions 19 and 20 of the *K-RAS *sequence. Sequence trace from a bacterial clone (lower panel) showing the appearance of the double mutation L19F/T20A in the same allele. For cloning of tumour derived PCR amplicons, products were ligated into TOPOBlunt plasmids using ZeroBlunt TOPO cloning kits (Invitrogen, Paisley, UK) according to the manufacturer's instructions. Bacterial DNA was then isolated using Miniprep kits (Qiagen, Crawley, UK) according to the manufacturer's instructions and sequenced using M13 universal primers on a ABI3730xl Platform sequencer (Applied Biosystems, Warrington, UK).

Sequencing of exons 1 and 2 of *K-RAS *in a panel 186 colorectal adenocarcinoma samples, obtained as part of the European Prospective Investigation into Cancer and Nutrition (EPIC) Norfolk study cohort, identified 41 cancers harbouring mutations in *K-RAS *[16]. Forty of these mutations were located in codons 12 or 13, however, one sample demonstrated the presence of double mutant peaks, one at the third position of codon 19 (G > T giving rise to a leucine to phenylalanine (L19F) amino acid change) and the other at the first position of codon 20 (A > G giving rise to a threonine to alanine (T20A) change) (Figure 1). In order to assess if both base changes were present in the same allele we cloned and sequenced individual PCR amplicons of *K-RAS *exon 1 harbouring the L19F/T20A mutations. Analysis of sequencing traces from individual bacterial clones identified solely double mutant (L19F/T20A) alleles (Figure 1), thus confirming the presence of both mutations on the same allele. In order to confirm the somatic nature of both elements of this co-mutation, non-cancerous DNA from the blood of the same individual was sequenced. Blood samples were obtained upon the volunteer's enrolment into the initial EPIC study. Following tumour development, both blood and tumour tissue were made available for analysis and processed for DNA extraction [16]. Sequencing of *K-RAS *exon 1 in blood DNA demonstrated only the presence of wildtype alleles, confirming that neither of the DNA sequence changes giving rise to the L19F or the T20A amino acid changes were germline polymorphisms.

Missense mutations of *K-RAS *codon 20 in human cancers have not been previously reported, however, mutations giving rise to phenylalanine incorporation into *K-RAS *codon 19 have been described previously in seven individual human colorectal cancers, a single human lung adenocarcinoma sample and a single lymphoblastic leukaemia [17-20]. No observations of a double mutation giving rise to both L19F and T20A amino acid alterations have been described previously. As such, the observation in our study of double mutant peaks giving rise to L19F/T20A missense co-mutations at codons 19 and 20 of *K-RAS *describes a previously unreported change in this gene.

**Figure 2:**
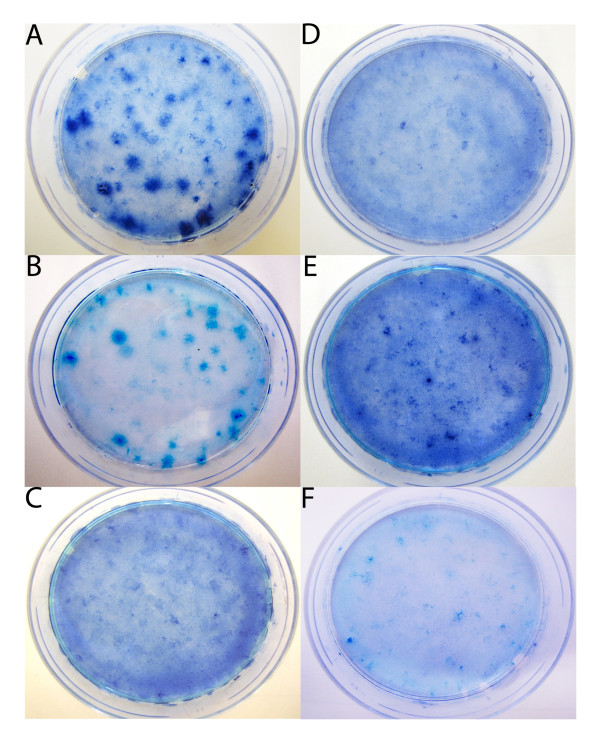
**Focus formation by mutated *K-RAS *cDNA**. Methylene blue stained transformed foci following transfection of *K-RAS *cDNA expression vectors containing: (A) G12D (B) G12V (C) L19F alone (D) T20A alone (E) L19F/T20A co-mutation and (F) wildtype sequences. "No DNA" controls describe cells treated with transfection reagents but no DNA. Colonies formed following transfection with the L19F/T20A double mutant *K-RAS *cDNA were consistently smaller than those formed following transfection with the codon 12 mutants. *K-RAS *cDNA containing plasmids were lipofected with Lipofectamine (Invitrogen, Paisley, UK) into NIH3T3 mouse fibroblast cells, and seeded onto 60 mm plates at a density of 100 000 cells per plate. The cells were grown for 14 days, with the media replaced every 72 hours. After 14 days plates were fixed in 10% formalin then stained with 1% methylene blue (Sigma-Aldrich, Gillingham, UK), in 70% ethanol solution. Colonies above 3 mm in diameter were counted and were statistically analysed using Mann-Whitney U tests with P values of less than or equal to 0.05 considered statistically significant.

**Figure 3:**
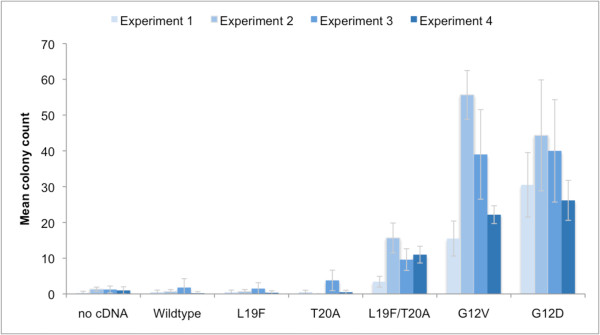
**Quantification of focus formation by different *K-RAS *mutations**. Histogram of mean focus numbers obtained from focus formation assays performed in 4 separate experiments after NIH3T3 cells were transfected with *K-RAS *cDNA expression vectors containing G12V, G12D, L19F, T20A, L19F/T20A or wildtype *K-RAS *sequences. Error bars represent ±SD. Full focus counts for all transfections and all experiments and the sequences of the cDNA constructs used for NIH3T3 cell transfection are described in Additional File section (Additional File 1).

A series of full length human *K-RAS *isoform *B *cDNA sequences were designed and synthesised containing either G12V, G12D, L19F, T20A, combined L19F/T20A or wildtype sequences and these were cloned into pBABE-puro expression vector plasmids for transfection into mouse NIH3T3 cells for focus forming assays (see Additional File 1). Transfection of *K-RAS *cDNA constructs containing individual L19F or T20A mutations did not show significantly increased transformation above that of the control cells, which were treated with either transfection reagents but no cDNA or transfection reagents and wildtype *K-RAS *cDNA. However, cells transfected with constructs containing the double mutation L19F/T20A in *K-RAS *cDNA demonstrated significantly increased focus formation above that of wildtype *K-RAS *cDNA transfected control cells (Mann-Whitney U test: *P *= 0.03; Figures 2 and 3). Additionally, the *K-RAS *L19F/T20A co-mutation was shown to form fewer foci than cells transfected with either G12D or G12V mutated *K-RAS *cDNA (*P *= 0.02 and 0.04 respectively). These data demonstrated that this L19F/T20A co-mutation of *K-RAS *confers oncogenic activation capable of inducing transformed focus formation to a greater degree than single mutations at either codons 19 or 20 or wildtype *K-RAS *cDNA, but to a lesser degree than codon 12 mutants of *K-RAS*.

It is not surprising that the T20A substitution alone was not sufficiently oncogenic to cause focus formation as this amino acid alteration has never been found previously in human cancers. However, the L19F substitution has been documented, albeit in a limited number of studies, and may be expected to have some transforming potential. One study described analysis of L19F in the *C. elegans RAS *homologue *let-60*. *C. elegans *carrying this mutation showed a temperature-sensitive multivulval phenotype. In a mammalian system, at body temperature, H-RAS (L19F) protein had a reduced rate of GTP hydrolysis relative to wildtype H-RAS, suggesting that H-RAS L19F conferred an increased level of activation [21]. However, transfection of NIH3T3 cells with human *H-RAS *with the incorporated L19F mutation failed to demonstrate increased focus formation above that of controls, a similar observation to that made here using L19F *K-RAS*. A second report, however, describes L19F as causing increased cell proliferation, anchorage-independent growth, increased tumourigenicity in nude mice and elevated levels of RAS-GTP [17]. Additionally, a recent analysis describing *K-RAS *mutations outside of codons 12 and 13 also described mildly increased focus formation by L19F mutants [22]. In this report the L19F *K-RAS *mutation was described to induce formation of ~5 foci whereas positive controls (G12V and G12D) were observed to develop ~80 - 90 foci. These data are not mutually exclusive with our observations, as the mean focus count observed here with the G12D mutant was 35 colonies per plate, thus a mild focus forming ability of the L19F mutation may not have been detected. The observed differences between the number of colonies formed in the study by Smith and colleagues [22] and those in our own assays may be due to variations in protocols used, such as the different expression vectors used in the two analyses giving rise to different levels of transcription following transfection into NIH3T3 cells and different tissue culture plating conditions.

Our identification and analysis of the L19F/T20A double mutation in *K-RAS *is the first description of this genetic change, which confers a greater transforming ability than individual mutations in these codons. The rare observed frequency of this double amino acid change giving rise to oncogenic *K-RAS *in human cancers, compared with that of the individual L19F amino acid change, despite its increased oncogenic potential, may be due to the lower likelihood of 2 base changes occurring at these positions in the same allele during cancer development.

**Figure 4:**
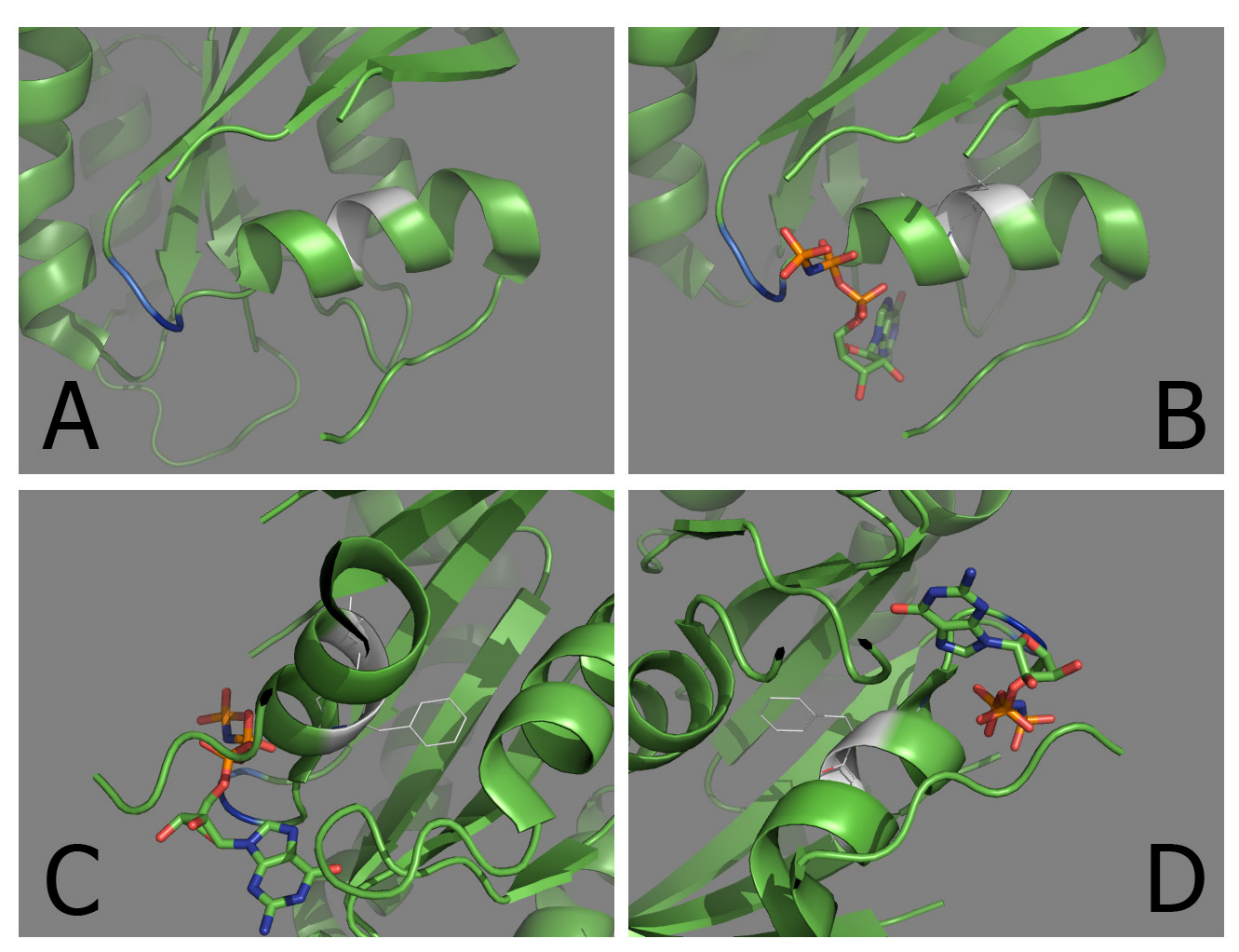
**Modelling of the mutant codon 19 phenylalanine and codon 20 alanine side chains into the K-RAS protein**. (A) The GDP/GTP binding pocket of wildtype K-RAS. The white residues are codons 19 and 20. The loop on the left of the helix forms the GDP/GTP binding pocket of K-RAS. Residues coloured blue represent the mutational hotspot codons 12 and 13. The shortened K-RAS protein structure used for modelling was obtained from the Protein Data Bank (PDB ID:3gft) and visualised using PyMOL (http://www.pymol.org). (B) The GDP/GTP binding pocket of wildtype K-RAS with wildtype side chains of codons 19 and 20 and a non-hydrolysable GTP analogue (used during crystallisation) adjacent to codons 12 and 13. (C and D) Modelling of the mutant phenylalanine (codon 19) and alanine (codon 20) side chains into the K-RAS protein, predicts projection of the bulky phenylalanine side chain into the main body of the protein and replacement of the OH containing side chain of threonine with the small aliphatic side chain of alanine. The mutant side chains were modelled into positions of the protein which produced the fewest conflicting Van der Waals radii and the configuration with the least steric interference with other amino acid side chains.

To assess the potential structural effects of the L19F/T20A amino acid changes we modelled their side chains into a shortened wildtype K-RAS protein ribbon structure bound to a GTP analogue (Figure 4a). Codons 19 and 20 (shown in white in Figure 4b) are in close proximity to the GDP/GTP binding pocket of the protein. Modelling of the mutant phenylalanine (codon 19) and alanine (codon 20) side chains into the wildtype protein predicted two features. First, the removal of the leucine side chain at codon 19 and its replacement with that of phenylalanine resulted in projection of the bulky, ringed side chain of the newly encoded amino acid into the main body of the protein and this may be predicted to change the shape of the GDP/GTP pocket by steric interference. Second, removal of the threonine side chain at codon 20, with loss of its OH group and its replacement with alanine that has a smaller aliphatic side chain may be predicted to affect the ionic interactions between nearby amino acid side chain groups (Figure 4c &4d). The presence of changes at both codons 19 and 20, demonstrating a higher transforming activity than individual mutations in these codons, suggests that shifting of the protein conformation due to steric interference together with disruption of ionic interactions within the K-RAS protein, may be responsible for generating a protein configuration capable of providing the observed transforming activity, by interfering with the geometry of the GDP/GTP binding pocket reducing GTP hydrolysis. Further structural studies are required to test this proposed mechanism.

*K-RAS *mutations affecting codons 15 and 22 have also been reported in human colorectal cancers [23,24]. Although the transforming ability of these specific codon changes is yet to be confirmed, the observation of sequence changes in human cancers affecting codons 15 and 22, as well as at co-mutation of codons 19 and 20, as described here, strongly suggests that alteration of this region in the K-RAS protein provides a selective growth advantage for cells which contributes to neoplastic transformation.

## Competing interests

The authors declare that they have no competing interests.

## Authors' contributions

All authors contributed to the study design, analyses and manuscript preparation. All authors read and approved the final manuscript.

## Supplementary Material

Additional file 1 **Experimental methods and statistical testing**. Additional File 1 contains a detailed description of all experimental methods described in this manuscript. Furthermore, complete colony counts are available for all experiments in tabulated format. Click here for file

## References

[B1] Downward J Targeting RAS signalling pathways in cancer therapy Nat Rev Cancer 2003 3 11 22 10.1038/nrc969 12509763

[B2] Bos JL ras oncogenes in human cancer: a review Cancer Res 1989 49 4682 4689 2547513

[B3] Nishimura S Sekiya T Human cancer and cellular oncogenes Biochem J 1987 243 313 327 3307760 10.1042/bj2430313 PMC1147857

[B4] Edkins S O'Meara S Parker A Stevens C Reis M Jones S Greenman C Davies H Dalgliesh G Forbes S Recurrent KRAS codon 146 mutations in human colorectal cancer Cancer Biol Ther 2006 5 928 932 10.4161/cbt.5.8.3251 16969076 PMC2714972

[B5] Seeburg PH Colby WW Capon DJ Goeddel DV Levinson AD Biological properties of human c-Ha-ras1 genes mutated at codon 12 Nature 1984 312 71 75 10.1038/312071a0 6092966

[B6] Andreyev HJ Norman AR Cunningham D Oates JR Clarke PA Kirsten ras mutations in patients with colorectal cancer: the multicenter "RASCAL" study J Natl Cancer Inst 1998 90 675 684 10.1093/jnci/90.9.675 9586664

[B7] Malumbres M Barbacid M RAS oncogenes: the first 30 years Nat Rev Cancer 2003 3 459 465 10.1038/nrc1097 12778136

[B8] Arends MJ McGregor AH Toft NJ Brown EJ Wyllie AH Susceptibility to apoptosis is differentially regulated by c-myc and mutated Ha-ras oncogenes and is associated with endonuclease availability Br J Cancer 1993 68 1127 1133 10.1038/bjc.1993.492 8260364 PMC1968632

[B9] Arends MJ McGregor AH Wyllie AH Apoptosis is inversely related to necrosis and determines net growth in tumors bearing constitutively expressed myc, ras, and HPV oncogenes Am J Pathol 1994 144 1045 1057 8178928 PMC1887373

[B10] Spandidos DA Wilkie NM Malignant transformation of early passage rodent cells by a single mutated human oncogene Nature 1984 310 469 475 10.1038/310469a0 6462235

[B11] Brooks DG James RM Patek CE Williamson J Arends MJ Mutant K-ras enhances apoptosis in embryonic stem cells in combination with DNA damage and is associated with increased levels of p19(ARF) Oncogene 2001 20 2144 2152 10.1038/sj.onc.1204309 11360198

[B12] Luo F Brooks DG Ye H Hamoudi R Poulogiannis G Patek CE Winton DJ Arends MJ Conditional expression of mutated K-ras accelerates intestinal tumorigenesis in Msh2-deficient mice Oncogene 2007 26 4415 4427 10.1038/sj.onc.1210231 17297472

[B13] Luo F Brooks DG Ye H Hamoudi R Poulogiannis G Patek CE Winton DJ Arends MJ Mutated K-ras(Asp12) promotes tumourigenesis in Apc(Min) mice more in the large than the small intestines, with synergistic effects between K-ras and Wnt pathways Int J Exp Pathol 2009 90 558 574 10.1111/j.1365-2613.2009.00667.x 19765110 PMC2768154

[B14] Luo F Hamoudi R Brooks DG Patek CE Arends MJ Stem cell gene expression changes induced specifically by mutated K-ras Gene Expr 2007 14 101 115 10.3727/105221607783417583 18257393 PMC6042043

[B15] Luo F Ye H Hamoudi R Dong G Zhang W Patek CE Poulogiannis G Arends MJ K-ras exon 4A has a tumour suppressor effect on carcinogen-induced murine colonic adenoma formation J Pathol 2010 220 542 550 10.1002/path.2672 20087880

[B16] Naguib A Mitrou PN Gay LJ Cooke JC Luben RN Ball RY McTaggart A Arends MJ Rodwell SA Dietary, lifestyle and clinicopathological factors associated with BRAF and K-ras mutations arising in distinct subsets of colorectal cancers in the EPIC Norfolk study BMC Cancer 2010 10 99 10.1186/1471-2407-10-99 20233436 PMC2847960

[B17] Akagi K Uchibori R Yamaguchi K Kurosawa K Tanaka Y Kozu T Characterization of a novel oncogenic K-ras mutation in colon cancer Biochem Biophys Res Commun 2007 352 728 732 10.1016/j.bbrc.2006.11.091 17150185

[B18] Ferraz JM Zinzindohoue F Lecomte T Cugnenc PH Loriot MA Beaune P Stucker I Berger A Laurent-Puig P Impact of GSTT1, GSTM1, GSTP1 and NAT2 genotypes on KRAS2 and TP53 gene mutations in colorectal cancer Int J Cancer 2004 110 183 187 10.1002/ijc.20124 15069679

[B19] Lin JK Chang SC Wang HS Yang SH Jiang JK Chen WC Lin TC Li AF Distinctive clinicopathological features of Ki-ras mutated colorectal cancers J Surg Oncol 2006 94 234 241 10.1002/jso.20438 16900509

[B20] Simi L Pratesi N Vignoli M Sestini R Cianchi F Valanzano R Nobili S Mini E Pazzagli M Orlando C High-resolution melting analysis for rapid detection of KRAS, BRAF, and PIK3CA gene mutations in colorectal cancer Am J Clin Pathol 2008 130 247 253 10.1309/LWDY1AXHXUULNVHQ 18628094

[B21] Eisenmann DM Kim SK Mechanism of activation of the Caenorhabditis elegans ras homologue let-60 by a novel, temperature-sensitive, gain-of-function mutation Genetics 1997 146 553 565 9178006 10.1093/genetics/146.2.553 PMC1207997

[B22] Smith G Bounds R Wolf H Steele RJ Carey FA Wolf CR Activating K-Ras mutations outwith 'hotspot' codons in sporadic colorectal tumours - implications for personalised cancer medicine Br J Cancer 2010 102 693 703 10.1038/sj.bjc.6605534 20147967 PMC2837563

[B23] Miyakura Y Sugano K Fukayama N Konishi F Nagai H Concurrent mutations of K-ras oncogene at codons 12 and 22 in colon cancer Jpn J Clin Oncol 2002 32 219 221 10.1093/jjco/hyf043 12110640

[B24] Wang JY Hsieh JS Chen FM Yeh CS Alexandersen K Huang TJ Chen D Lin SR High frequency of activated K-ras codon 15 mutant in colorectal carcinomas from Taiwanese patients Int J Cancer 2003 107 387 393 10.1002/ijc.11417 14506738

