# Supplementary material for: Activation of K-RAS by co-mutation of codons 19 and 20 is transforming
Source: J Mol Signal. 2011 Mar 3;6:2. doi: 10.1186/1750-2187-6-2 (PMC3056876; doi:10.1186/1750-2187-6-2)
Supplement: Additional file 1 — Experimental methods and statistical testing. Additional File 1 contains a detailed description of all experimental methods described in this manuscript. Furthermore, complete colony counts are available for all experiments in tabulated format. [file 1750-2187-6-2-S1.DOC]

**Supplementary information**

**cDNA constructs used for transfection of NIH3T3 cells**

cDNA constructs were designed, synthesised *in vitro* (ordered from Geneart, Regensburg, Germany), cloned into expression vectors and transfected into NIH3T3 cells (ECACC, Salisbury, UK). Six *K-RAS* cDNA constructs were produced, transfected and studied. Two splice variants of *K-RAS* mRNA are generated in human and other mammalian cells, isoforms *A* and *B*, with the *B* isoform being more abundantly expressed . Consequently, the sequence of the *B* isoform was used for cDNA construction. Wildtype sequence was obtained from previously published analyses and confirmed by comparison with reference sequences using the Ensembl Genome Browser (www.ensembl.org). cDNA inserts were designed containing *BamH*I (GGATCC) and *Sal*I (GTCGAC) restriction sites at the 5’ and 3’ ends respectively. Immediately after the *BamH*I site a Kozak sequence (GCCGCCACC) was inserted to facilitate ribosome binding at the translation stage.

Upon receipt from the manufacturer, cDNA constructs were solubilised to a concentration of 200 ng/µl. Subsequently, each of the cDNAs was digested in preparation for insertion, cloning and ligation into pBABE-puro expression vector plasmids (Addgene, Cambridge, USA). The reaction mixtures were as follows: 5 µl cDNA (200 ng/µl), 5 µl x10 Buffer3, 1 µl *BamH*I (10,000 U/µl), 1 µl *Sal*I (20,000 U/µl) (all New England Biolabs, Ipswich, USA) made to a total volume of 50 µl with nuclease free water (Promega, Madison, USA). The plasmid vector was also digested in preparation for cDNA insertion. The reaction mixture was as for the cDNA inserts with the plasmid DNA being at a higher starting concentration of 695 ng/µl. The reactions were incubated at 37°C for 1 hour. The 50 µl digested DNA mixture was then run on a 1% agarose gel containing 1 µg/ml ethidium bromide and visualised using UV light. Successful digestion was confirmed due to identification of correct size of the restriction fragments (582 bp for cDNA inserts and 5127 bp for the plasmid). The correct size bands were then excised from the gel and incubated at 70°C for 10 minutes in order to melt the agarose. The samples were then cleaned using Qiaquick spin columns (Qiagen, Crawley, UK) according to the manufacturer's instructions and eluted to a final volume of 20 µl in 10 mM Tris. Ligation of the digested cDNA fragments into the cut plasmid was performed in the following reaction mixture: 3 µl plasmid DNA solution, 5 µl cDNA insert, 1 µl DNA ligase , 1 µl x10 Buffer (both New England Biolabs, Ipswich, USA). Following ligation, the plasmids with ligated insert were electroporated at 1.8 kV with 5 µl of electrocompetent DH10β *E. coli* bacterial cells (DSMZ, Braunschweig, Germany), and then spread onto lysogeny broth agar plates (containing 0.1% ampicillin and 0.1% β –galactosidase) and incubated at 37°C for 12 hours. Colonies demonstrating successful transfection were then picked and grown individually, 8 replicates per cDNA insert type. Bacterial DNA was then isolated using Qiagen miniprep kits (Qiagen, Crawley, UK) according to the manufacturer's instructions. Following DNA isolation, in order to confirm correct insertion of the *K-RAS* cDNA into the plasmid, digestion of each sample was performed. Each construct underwent a single and a double digestion. The single digestion, using *Hind*III in a reaction mixture as described above except that the buffer used was x10 Buffer2 (New England Biolabs, Ipswich, USA), generated a single 5709 bp product for successfully inserted plasmids. The double digestion, using *BamH*I and *Sal*I, generated 586 bp and 5123 bp fragments from successful plasmid inserts. Correct insertion was also confirmed by didoxysequencing. Primers were designed to anneal to the regions of the plasmid flanking the insert: F:5' -CCT CCC TTT ATC CAG CCC TCA CTC CTT CTC TAG GCG CC-3' , R:5' -CCT GGG GAC TTT CCA CAC CCT AAC TGA CAC AC-3'. PCR products were generated using 1 µg of sample DNA: concentrations were determined prior to PCR using a Nanodrop ND-1000 Spectrophotometer (Labtech International Ltd, Ringmer, UK) . KOD Hot Start DNA Polymerase kits (Novagen, Madison, USA) were used to make the following reaction mixture: 2.5 µl x10 PCR Buﬀer for KOD Hot Start DNA Polymerase, 1 µl primers, forward and reverse (10 µM each), 1 µl

MgSO_4_ (25 mM), 2 µl dNTPs (2 mM each), 0.25 µl KOD DNA Polymerase and made up to a total reaction volume of 25 µl in nuclease-free water (Promega, Madison, USA). PCR reactions involved a denaturation step at 94°C for 5 minutes followed by 45 cycles of 94°C for 15 seconds, 30 seconds at an annealing temperature of 55°C and then 72°C for 30 seconds. The ﬁnal extension step was 72°C for 5 minutes. Reactions were performed using an MJ Research PTC-225 Peltier Thermal Cycler (BioRad, Hertfordshire, UK). Dideoxysequencing was performed on an ABI3730xl Platform sequencer (Applied Biosystems, Warrington, UK). The PCR primers were used as sequencing primers. All sequencing confirmed the correct *K-RAS* cDNA insertion and orientation. Following identification of successfully inserted clones, bacterial clones confirmed as containing ligated plasmid were then grown for each *K-RAS* insert type, and further bacterial plasmid DNA was isolated as described above and used for NIH3T3 mouse fibroblast transfection.

**Statistical testing**

Focus counts were assessed for statistically significant differences using non-parametric testing (Mann-Whitney U), with test formats hypothesis driven. Our original hypothesis that the double L19F/T20A mutation, identified in a human cancer sample, was oncogenic, indicated that a one-tailed Mann-Whitney U test was to be performed when analysing if this genetic change induced formation of more foci than wildtype *K-RAS* sequence. No prior hypothesis regarding the comparison of the double mutation and the codon 12 mutants was formed. As such, in order to assess the difference between the double mutation and the codon 12 mutants in terms of focus formation, a two-tailed test was performed. Focus counts were performed openly.

Table S1: Descriptions of the deviations from wildtype of *K-RAS* cDNA used for transfections. The wildtype *K-RAS* cDNA was isoform *B.*

| **cDNA name** | **Deviations from wildtype *K-RAS*** | **Base and residue changes** |
| --- | --- | --- |
| *K-RAS* wildtype  *K-RAS* G12V | None | None: *K-RAS* wildtype cDNA |
|  | Codon 12 | GGT⇒GTT (Gly. ⇒Val.) |
| *K-RAS* G12D | Codon 12 | GGT⇒GAT (Gly. ⇒Asp.) |
| *K-RAS* L19F | Codon 19 | TTG⇒TTT (Leu. ⇒Phe.) |
| *K-RAS* T20A | Codon 20 | ACG⇒GCG (Thr. ⇒Ala.) |
| *KRAS* L19F & T20A | Codons 19 and 20 | TTG⇒TTT (Leu. ⇒Phe.) and ACG⇒GCG (Thr. ⇒Ala.) |

Table S2: Focus formation counts in NIH3T3 cells transfected with *K-RAS* expression constructs including the codon 19 and 20 base changes observed in one EPIC colorectal adenocarcinoma and other oncogenic *K-RAS* mutations. The results of four focus formation assays are displayed. ‘No cDNA’ describes transfection using Lipofectamine reagents with no construct DNA. Focus formation counts represent individual, unique plates; the mean focus count being derived from these combined data. Experiments 1-4 represent 4 individual biological experiment repeats.

|  | **Colony counts** | **Mean colony count** |
| --- | --- | --- |
| **Experiment 1**  *K-RAS* G12V |  |  |
|  | 15, 23, 19, 11, 10, 15 | 16 |
| *K-RAS* G12D | 27, 34, 24, 18, 39, 41 | 31 |
| *K-RAS* L19F | 0, 0, 1, 1 | 1 |
| *K-RAS* T20A | 0, 0, 0, 1, 1, 1 | 1 |
| *K-RAS* L19F and T20A | 1, 5, 3, 2, 5, 4, 4 | 3 |
| Wildtype *K-RAS* | 0, 0, 1, 1 | 1 |
| No cDNA | 0, 0, 0, 1 | 0 |

|  | **Colony counts** | **Mean colony count** |
| --- | --- | --- |
| **Experiment 2**  *K-RAS* G12V |  |  |
|  | 48, 58, 61 | 56 |
| *K-RAS* G12D | 29, 44, 60 | 44 |
| *K-RAS* L19F | 0, 1, 1 | 1 |
| *K-RAS* T20A | 0, 0, 0 | 0 |
| *K-RAS* L19F and T20A | 11, 17, 19 | 16 |
| Wildtype *K-RAS* | 0, 1, 1 | 1 |
| No cDNA | 1, 1, 2 | 1 |

|  | **Colony counts** | **Mean colony count** |
| --- | --- | --- |
| **Experiment 3**  *K-RAS* G12V |  |  |
|  | 30, 31, 38, 57 | 39 |
| *K-RAS* G12D | 21, 37, 50, 52 | 40 |
| *K-RAS* L19F | 0, 0, 1, 1, 3, 4 | 2 |
| *K-RAS* T20A | 0, 2, 4, 6, 7 | 4 |
| *K-RAS* L19F and T20A | 6, 8, 9, 11, 14 | 10 |
| Wildtype *K-RAS* | 0, 0, 1, 2, 6 | 2 |
| No cDNA | 0, 1, 2, 2 | 1 |

|  | **Colony counts** | **Mean colony count** |
| --- | --- | --- |
| **Experiment 4**  *K-RAS* G12V |  |  |
|  | 18, 21, 22, 23, 24, 25 | 22 |
| *K-RAS* G12D | 19, 23, 25, 25, 30, 35 | 26 |
| *K-RAS* L19F | 0, 0, 1 | 0 |
| *K-RAS* T20A | 0, 0, 0, 1, 1, 1 | 1 |
| *K-RAS* L19F and T20A | 9, 10, 10, 11, 15 | 11 |
| Wildtype *K-RAS* | 0, 0, 0, 0, 1 | 0 |
| No cDNA | 0, 1, 2 | 1 |

*References*

1. Bar-Sagi D: **A Ras by any other name.** *Mol Cell Biol* 2001, **21:**1441-1443.

2. Capon DJ, Seeburg PH, McGrath JP, Hayflick JS, Edman U, Levinson AD, Goeddel DV: **Activation of Ki-ras2 gene in human colon and lung carcinomas by two different point mutations.** *Nature* 1983, **304:**507-513.
